# NG2 as an Identity and Quality Marker of Mesenchymal Stem Cell Extracellular Vesicles

**DOI:** 10.3390/cells8121524

**Published:** 2019-11-27

**Authors:** Mario Barilani, Valeria Peli, Alessandro Cherubini, Marta Dossena, Vincenza Dolo, Lorenza Lazzari

**Affiliations:** 1 Laboratory of Regenerative Medicine–Cell Factory, Department of Transfusion Medicine and Hematology, Fondazione IRCCS Cà Granda Ospedale Maggiore Policlinico, via F. Sforza 35, 20122 Milano (MI), Italy; mario.barilani@policlinico.mi.it (M.B.); valeria.peli@live.com (V.P.); alessandro.cherubini@policlinico.mi.it (A.C.); marta.dossena@policlinico.mi.it (M.D.); 2 Department of Life, Health and Environmental Sciences, University of L’Aquila, 67100 L’Aquila, Italy; vincenza.dolo@univaq.it

**Keywords:** exosomes, microvesicles, neural/glial antigen 2, chondroitin sulfate proteoglycan 4, CSPG4, melanoma-associated chondroitin sulfate proteoglycan, MCSP, cord blood, bone marrow, MSC

## Abstract

The therapeutic potential of mesenchymal stem cell (MSC) extracellular vesicles (EV) is currently under investigation in many pathological contexts. Both adult and perinatal MSC are being considered as sources of EV. Herein, we address antigen expression of cord blood and bone marrow MSC and released EV to define an identity and quality parameter of MSC EV as a medicinal product in the context of clinical applications. The research focuses on EV-shuttled neural/glial antigen 2 (NG2), which has previously been detected as a promising surface marker to distinguish perinatal versus adult MSC. Indeed, NG2 was significantly more abundant in cord blood than bone marrow MSC and MSC EV. Ultracentrifuge-isolated EV were then challenged for their pro-angiogenic properties on an xCELLigence system as quality control. NG2^+^ cord blood MSC EV, but not bone marrow MSC EV, promote bFGF and PDGF-AA proliferative effect on endothelial cells. Likewise, they successfully rescue angiostatin-induced endothelial cell growth arrest. In both cases, the effects are NG2-dependent. These results point at NG2 as an identity and quality parameter for cord blood MSC EV, paving the way for their clinical translation.

## 1. Introduction

In the last few years, a remarkable shift in the mesenchymal stem cell (MSC) field has occurred [1]. The focus of the researchers has switched from the differentiation and growth factor-secreting properties of MSC to the therapeutic potential of their extracellular vesicles (EV) [2]. This term was adopted by the International Society for Extracellular Vesicles (ISEV) [3,4] to comprise the different class of membranous vesicles secreted by virtually all cell types into the extracellular environment. The most studied among them are exosomes and microvesicles. The former of endosomal origin, with a size roughly ranging from 40 to 150 nm [5], the latter budding from the plasma membrane, with a broader size range of 100–1000 nm [6]. In particular, EV from both adult [7] and perinatal MSC [8] have drawn attention for their immunomodulatory functions and regenerative capacity [9,10,11,12,13]. Based on these features, MSC EV are currently under investigation in many pathological contexts. Similarly to cell therapies, translation to the clinical application of vesicular therapies entails the definition of clinical-grade identity parameters and the development of rapid and simple assays indicative of retention of biological activity after production and storage. These tests will be needed to guarantee the quality of EV-based therapeutics and to ensure consistent outcomes in different clinical centers [14,15]. Nevertheless, specific markers for EV of diverse mesenchymal origin are still lacking, as well as EV preparations quality assays, whereas more effort is dedicated to the exploration of functional properties and fields of therapeutic application. 

In a previous study, we addressed the expression of stemness-associated surface antigens in perinatal and adult MSC [16] and evidenced neuron-glial antigen 2 (NG2) as a promising candidate marker of perinatal MSC versus adult MSC. This type 1 membrane protein is highly expressed by pericytes [17], which were indicated as the in vivo progenitors of MSC [18], and has a prominent role in the support of angiogenesis [19,20]. In detail, an NG2 knockout resulted in significant deficits in blood vessel formation during development, but also in tumor vascularization in adult organisms. In the present study, we want to investigate if EV released by perinatal and adult MSC preserve the parental cell differential expression of the NG2 antigen. Furthermore, we want to test whether NG2 can be used as an identity and quality marker for perinatal MSC. To this aim, long-living cord blood MSC (LL-cbMSC) and bone marrow MSC (bmMSC) were used, as representative of perinatal and adult MSC populations, respectively. NG2 expression was observed and validated in parental cells and generated EV. The abundance of the antigen was remarkably higher in LL-cbMSC than bmMSC. Intriguingly, the difference was maintained in the secreted EV. Furthermore, we challenged this EV-shuttled antigen in a potency assay experimental setting. LL-cbMSC but not bmMSC EV showed remarkable NG2-dependent pro-angiogenic properties, which demonstrated the functional relevance of the NG2 dose and the importance of the EV MSC source type.

## 2. Materials and Methods

### 2.1. Reagents 

The following reagents were used in this study, listed in alphabetical order: αMEM-GlutaMAX (32571028; Thermo Fisher Scientific, Waltham, MA, USA); angiostatin (4919-100; BioVision, Milpitas, CA, USA); bFGF (RFGFB50; Thermo Fisher Scientific); CellTrace CFSE Cell Proliferation Kit (C34554; Thermo Fisher Scientific); DAPI (10236276001; Roche, Basel, Switzerland); DMEM without phenol red (31053028; Thermo Fisher Scientific); donkey anti-rabbit IgG HRP-linked secondary antibody (NA934-1ML; GE Healthcare, Chicago, IL, USA); endomed medium (F2245; Biochrome, Berlin, Germany); fetal bovine serum (FBS, 10270; Thermo Fisher Scientific); glutaraldehyde (G-5882; Sigma-Aldrich, Saint Louis, MI, USA); goat anti-mouse IgG HRP-linked secondary antibody (170-6516; Bio-Rad, Hercules, CA, USA); goat anti-rabbit IgG AF568-conjugated secondary antibody (A11011; Thermo Fisher Scientific); mouse anti-human β-actin antibody (A5441; Sigma-Aldrich); mouse anti-human CD63 antibody (CBL553; Millipore, Burlington, MA, USA); mouse anti-human CD81 antibody (555675; BD, Franklin Lakes, NJ, USA); mouse anti-human PE-conjugated NG2 antibody (IM3454U; Beckman Coulter, Brea, CA, USA); mouse IgG1 PE-conjugated isotype control (A07796; Beckman Coulter); nonfat milk (Blotting-Grade Blocker, 1706404; Bio-Rad); normal goat serum (566380; Sigma-Aldrich); paraformaldehyde (15710; Electron Microscopy Sciences, Hatfiled, PA, USA); PBS (ECB4004L; Euroclone, Pero, Italy); PDGF-AA (100-13A; Peprotech, Rocky Hill, NJ, USA); Restore PLUS Western blot stripping buffer (46430; Thermo Fisher Scientific); TRIzol (15596-026; Thermo Fisher Scientific); Triton X-100 (T8787; Sigma-Aldrich); TrypLE Select enzyme (12563011; Thermo Fisher Scientific); rabbit anti-human NG2 antibody (sc-20162; Santa Cruz Biotechnology, Dallas, TX, USA); RIPA (R0278; Sigma-Aldrich); SuperScript IV VILO (11756500; Thermo Fisher Scientific); SYBR Select master mix (4472937; Thermo Fisher Scientific); Thiazolyl Blue tetrazolium bromide (M2128; Sigma-Aldrich).

### 2.2. Cell Culture

LL-cbMSC (*n* = 3) and bmMSC (*n* = 3) used in this work were randomly selected from mesenchymal cell samples previously obtained and fully characterized for appropriate MSC identity following the International Society for Cellular Therapy guidelines [21,22], as described in detail in recent publications [16,23,24,25,26]. LL-cbMSC were isolated from discarded cb units of healthy donors (at term 40 ± 2 weeks gestational age; *n* = 2 male donors, *n* = 1 female donor) not suitable for transplantation due to insufficient volume or white blood cell count, whereas bmMSC were isolated from healthy individuals undergoing bone fracture repair (51 ± 9 years old; *n* = 2 male donors, *n* = 1 female donor). Written informed consent was obtained from all donors involved in the research. No sensitive data of the donors were disclosed. The authors state that this study was performed according to the amended Declaration of Helsinki. Medium changes were performed twice a week with αMEM-GlutaMAX (Thermo Fisher Scientific) supplemented with 20% FBS (Thermo Fisher Scientific). Cell cultures were maintained at 37 °C, 5% CO^2^ in a humidified atmosphere. At 80% confluence, the cells were harvested using TrypLE Select enzyme (Thermo Fisher Scientific) and seeded at 4 × 10^3^ cells/cm^2^ in T175 cm^2^ flasks for expansion. 

### 2.3. Microculture Tetrazolium Assay 

Microculture tetrazolium (MTT) assay was performed as elsewhere described [24] to study MSC proliferation. Briefly, 4000 cells/cm^2^ were seeded into 96-well plates in quadruplicates and analysis was performed at 24, 48, 72, 96, 168 h time points. Thiazolyl Blue tetrazolium bromide (Sigma-Aldrich) was used at 0.5 mg/mL in DMEM without phenol red (Thermo Fisher Scientific) and the formazan crystals generated were dissolved in 96% ethanol. Optical density was measured at 570 nm after subtraction of 650 nm background on a GENios microplate reader (TECAN, Männedorf, Switzerland). Population doubling time was calculated as follows: PDT = [(t2 − t1) × log(2)]/[(log(A2) − log(A1)], where t1 and t2 are two time points of exponential growth, while A1 and A2 are the respective absorbance values normalized to 24 h time point.

### 2.4. Standard Flow Cytometry

Cell flow cytometry analysis was performed as elsewhere described [16]. Briefly, at least 100,000 cells were stained with 10 µL PE-conjugated NG2 antibody (Beckman Coulter) or mouse IgG1 PE-conjugated isotype control (Beckman Coulter) in a total volume of 200 μL of PBS (Sigma-Aldrich) for 20 min in the dark at room temperature (RT). Next, cells were washed with PBS (Sigma-Aldrich), centrifuged at 350× *g* for 7 min at RT, suspended in 300 μL of PBS (Sigma-Aldrich) and analyzed on a FACSCanto II cytometer (BD). At least 10,000 events were acquired and plotted against forward scatter (FSC)-height and FSC-area (FSC-A) to exclude cell doublets (P1 gate). P1 events were plotted against FSC-A and side scatter (SSC-A) to exclude debris and necrotic cells (P2 gate). Analytical data, including percentage of PE-positive events and mean fluorescence intensity (MFI) of P2-gated events, were analyzed in Excel. The MFI ratio was calculated as MFI (stained sample)/MFI (unstained sample).

### 2.5. qPCR

Total RNA was isolated from 100,000 LL-cbMSC (*n* = 3) and bmMSC (*n* = 3) using TRIzol (Thermo Fisher Scientific), and its quality and quantity were evaluated on a NanoDrop ND-1000 spectrophotometer (NanoDrop Technologies, Wilmington, DE, USA). For the qPCR assay, cDNA was synthesized from 500 ng of total RNA with SuperScript IV VILO (Thermo Fisher Scientific). Next, qPCR was carried out using SYBR Select master mix (Thermo Fisher Scientific), according to the manufacturer’s protocols, on a CFX96 thermal cycler (Bio-Rad). The relative expression levels of the selected targets were determined using the ΔΔC_t_ method and normalized to GAPDH mRNA levels. The primers were designed using the NCBI primer-BLAST tool (https://www.ncbi.nlm.nih.gov/). The primer sequences used in the qPCR analysis were as follows: forward 5′- AGGTGAAGGTCGGAGTCAAC-3′, reverse 5′-CCATGTAGTTGAGGTCAATGAAG-3′ for GAPDH; forward 5′-CCTTGCTGTGGCTGTGTCTT-3′, reverse 5′-GGACCCAGAGACCTTTGTTCTT-3′ for NG2.

### 2.6. Extracellular Vesicles

LL-cbMSC and bmMSC extracellular vesicles (EV) were isolated following the serial centrifugations protocol developed by Thery et al. [27] for cell culture supernatant, with slight modifications. To harvest EV, cell cultures were rinsed three times with PBS (Sigma-Aldrich) to remove FBS EV and then incubated with FBS-free αMEM-GlutaMAX (Thermo Fisher Scientific) for 24 h. A total of 270 mL of cell culture supernatant was collected. To obtain pre-cleared EV-containing cell culture supernatants, residual cells and apoptotic bodies were removed by serial centrifugations. First, the samples were centrifuged at 350× *g* for 10 min at RT, and the supernatant was collected. Second, the supernatant was centrifuged at 4700× *g* for 15 min at RT, and the supernatant was collected again. Pre-cleared samples thus obtained were analyzed by nanoparticle tracking analysis (see below) or extracellular vesicle flow cytometry (see below), immediately or after storage at 4 °C for up to 2 weeks. For other applications needing concentrated EV, pre-cleared samples were further processed immediately or after storage at 4 °C for up to 2 weeks. Samples were diluted 1:1 with 0.1 µm-filtered PBS (Sigma-Aldrich) and centrifuged at 100,000× *g* for 2 h at 4 °C on a Sorvall WX 80+ ultracentrifuge (75000080; Thermo Fisher Scientific), using a carbon fiber composite F37L-8x100 fixed rotor (096-087056; Thermo Fisher Scientific) and 70 mL polycarbonate tubes (010-1333; Thermo Fisher Scientific). To remove any contaminant protein aggregates generated by ultracentrifugation and obtain sufficiently pure EV preparations, EV pellets were suspended in 0.1 µm-filtered PBS (Sigma-Aldrich) and centrifuged again at 100,000× *g* for 1 h at 4 °C, using 10.4 mL polycarbonate tubes (010-1382; Thermo Fisher Scientific) and suspended in 200 μL of 0.1 µm-filtered PBS (Sigma-Aldrich). Concentrated EV preparations thus obtained were used for transmission electron microscopy analysis (see below), Western analysis (see below), or for the in vitro model of endothelial cell growth support (see below), immediately or after storage at 4 °C for up to 1 week. 

### 2.7. Western Analysis

Proteins were extracted from 1 × 10^6^ pelleted MSC or from concentrated EV preparations in RIPA buffer (Sigma-Aldrich) without centrifugation. Protein concentration was quantified using Pierce BCA Protein Assay Kit (23227; Thermo Fisher Scientific). For tetraspanins evaluation, 5 µg of cellular and vesicular proteins were separated on Novex WedgeWell 4–12% Tris-Glycine Gels (XP04122BOX; Thermo Fisher Scientific), under non-reducing conditions. Blotting was performed via the iBlot 2 gel transfer device (Thermo Fisher Scientific) using iBlot polyvinylidene difluoride (PVDF) regular stacks (Thermo Fisher Scientific). To detect β-actin in tetraspanin blots, the membranes were stripped 10 min at RT with Restore PLUS Western blot stripping buffer (46430; Thermo Fisher Scientific). To detect NG2 protein, cellular (40 μg) and vesicular (6 μg) proteins were separated on a 6% polyacrylamide gel and blotted onto a PVDF membrane overnight at 4 °C in wet conditions. Immunoblotting conditions for all Western analyses were as follows. Membranes were blocked with 5% nonfat milk (Bio-Rad) and incubated with primary antibodies as follows: anti-NG2 (Santa Cruz Biotechnology) at 1:500 dilution for 2 h at RT (cellular samples) or overnight at 4 °C (vesicular samples); anti-β-actin (Sigma-Aldrich) at 1:5000 dilution overnight at 4 °C; anti-CD63 (Millipore) at 1:100 dilution overnight at 4 °C; anti-CD81 (BD) at 1:250 dilution overnight at 4 °C. After incubation with donkey anti-rabbit IgG HRP-linked secondary antibody (GE Healthcare) at 1:3000 dilution, or goat anti-mouse IgG HRP-linked secondary antibody (Bio-Rad) at 1:3000 dilution, proteins of interest were visualized with the Amersham ECL Prime Western blotting detection kit (RPN2232; GE Healthcare) or SuperSignal West Femto trial kit for vesicular NG2 detection (34095; Thermo Fisher Scientific). Chemiluminescence images were obtained on a Chemidoc XRS+ system (Bio-Rad). Uncropped images of all membranes are shown in Supplementary Figure S2.

### 2.8. Immunofluorescence

Cells grown on coverslips were fixed with 4% paraformaldehyde (Electron Microscopy Sciences) in PBS (Sigma-Aldrich) for 20 min, permeabilized in 0.3% Triton X-100 (Sigma-Aldrich) for 15 min, washed three times with PBS (Sigma-Aldrich) and blocked with PBS (Sigma-Aldrich) containing 5% normal goat serum (Sigma-Aldrich) and 0.3% Triton X-100 (blocking buffer) for 1 h at RT. Cells were incubated overnight at 4 °C with primary antibody NG2 (Santa Cruz Biotechnology) diluted 1:50 in blocking buffer, washed three times with PBS (Sigma-Aldrich) and then incubated with goat anti-rabbit IgG AF568-conjugated secondary antibody (Thermo Fisher Scientific) diluted 1:1000 in blocking buffer for 1 h at RT. Finally, the nuclei were stained with DAPI (Roche) for 10 min. Images were acquired using a Leica TCS SP8 X confocal microscope with HCX PL APO 63x/1.4 objective. Confocal z-stacks were acquired with sections of 0.3 µm.

### 2.9. Nanoparticle Tracking Analysis

The total number and size distribution of EV was estimated by nanoparticle tracking analysis (NTA), performed on an NS300 NanoSight instrument (Malvern Panalytical, Malvern, UK). PBS-diluted pre-cleared EV-containing cell culture supernatants were analyzed, acquiring 5 videos of 60 s in flow mode. Camera level and dilution were adjusted so that all measurements complied with the target quality parameters of 20–120 particles/frame and 3–6 threshold range. Particle concentration and size distribution were calculated by NanoSight NTA 3.3 software (Malvern Panalytical). 

### 2.10. Extracellular Vesicle Flow Cytometry

EV flow cytometry analysis was performed as outlined in Ragni et al. [10]. Briefly, 50 μL of pre-cleared EV-containing cell culture supernatant was incubated or not with 10 μM CellTrace CFSE cell proliferation kit (Thermo Fisher Scientific) for 30 min in the dark at RT. Staining was stopped by adding 300 μL of PBS (Sigma-Aldrich), and EV were analyzed immediately on a FACSCantoII cytometer (BD), setting FSC-A and SSC-A on a logarithmic scale and the FSC-A threshold in the 200–350 range. Events were acquired in low-speed mode and plotted against FSC-A and SSC-A to clearly distinguish electronic noise from EV events (P1 gate). P1 gate-selected events were then plotted against SSC-A and FL1 channel signals to evaluate CFSE positivity compared to an unstained sample. 

### 2.11. Electron Microscopy

Electron microscopy analysis was performed as already described [10]. Briefly, for scanning electron microscopy (SEM) the cells were fixed in PBS (Sigma-Aldrich) 2% glutaraldehyde (Sigma-Aldrich) and dehydrated with ethanol solutions. Next, samples were sputter-coated with an SCD040 Balzer Sputterer (Oerlikon Balzers, Balzers, Liechtenstein). Samples were analyzed with an SEM Philips 505 microscope (Philips, Amsterdam, Netherlands). Transmission electron microscopy was performed on concentrated EV preparations resuspended in PBS (Sigma-Aldrich) to analyze their ultrastructural morphology. According to proper dilutions, the samples were adsorbed to 300 mesh carbon-coated copper grids (Electron Microscopy Sciences) for 5 min in a humidified chamber at room temperature. EVs on grids were then fixed in 2% glutaraldehyde (Sigma-Aldrich) in PBS for 10 min and then briefly rinsed in milli-Q water. Grids with adhered EV were examined with a Philips CM 100 transmission electron microscope TEM at 80 kV, after negative staining with 2% phosphotungstic acid, brought to pH 7.0 with NaOH. Images were captured by a Kodak digital camera.

### 2.12. Bead-Based Extracellular Vesicle Flow Cytometry

EV immunophenotyping was performed on pre-cleared EV-containing cell culture supernatants with the Human MACSPlex exosome kit (130-108-813; Miltenyi Biotec, Bergisch Gladbach, Germany), following the manufacturer’s instructions. The kit consists of a cocktail of 39 fluorescently labeled bead populations, each coated with an antibody specific for one out of 37 surface epitopes or with the appropriate antibody out of two isotype controls. Briefly, 120 μL of sample was incubated overnight on a tube rotating mixer at RT in the dark with antibody-coated MACSPlex Exosome Capture Beads. The following bead-linked antibodies included in the kit were used: HLA-DRDPDQ REA, CD105 mouse IgG1, CD49e mouseIgG2b, CD9 mouse IgG1, HLA-ABC REA, CD63 mouse IgG1k, CD81 REA, NG2 (MCSP) mIgG1, CD146 mouse IgG1, CD44 mouse IgG1, CD29 mouse IgG1k, CD45 mouse IgG2a, CD31 mouse IgG1, CD14 mouse IgG2a, REA control, mouse IgG1 control. After a washing step with MACSPlex Buffer, the samples were incubated for 1 h on a tube rotating mixer at RT in the dark with a MACSPlex exosome detection reagent cocktail, comprising APC-conjugated antibodies against CD9, CD63, and CD81. The formation of complexes between antigen-specific capture beads, EV, and the detection reagent was evaluated on a FACSCanto II cytometer (BD). Single beads were visualized plotting acquired events against FSC-A and SSC-A (P1 gate). P1 gate-selected beads were then plotted against FITC-channel and PE-channel signals to detect 39 bead populations. Next, each bead population was analyzed for the APC signal to determine antigen expression compared to appropriate isotype control. 

### 2.13. In Vitro Model of Endothelial Cell Growth Support

Human umbilical vein endothelial cells (HUVEC) (C2517A; Lonza, Basilea, Switzerland) were seeded at 10,000 cells/cm^2^ in endomed medium (F2245; Biochrome, Berlin, Germany) directly in the 96-wells of a RTCA E-plate (05232368001; ACEA Biosciences, San Diego, CA, USA) in the presence or not of concentrated LL-cbMSC or bmMSC EV preparations, 5 μg/mL angiostatin (BioVision), 40 ng/mL bFGF (Thermo Fisher Scientific), and 20 ng/mL PDGF-AA (Peprotech). The E-plate was loaded onto an RTCA SP xCELLigence system (ACEA Biosciences). The xCELLigence system estimates cell proliferation measuring cellular impedance thanks to microelectrodes fused at the bottom of the wells. The automatic output of the system is named the cell index parameter. The cultures were monitored in real-time during 48 h performing cell index calculation every hour. Cell index values were used to plot results. Where specified, concentrated MSC EV preparations were incubated with NG2 antibody (Beckman Coulter) for 1 h at RT, before administration to HUVEC. Untreated HUVEC were used as the internal control.

## 3. Results

### 3.1. NG2 is a Consistent Surface Marker of Cord Blood versus Bone Marrow Mesenchymal Stem Cells

**Figure 1:**
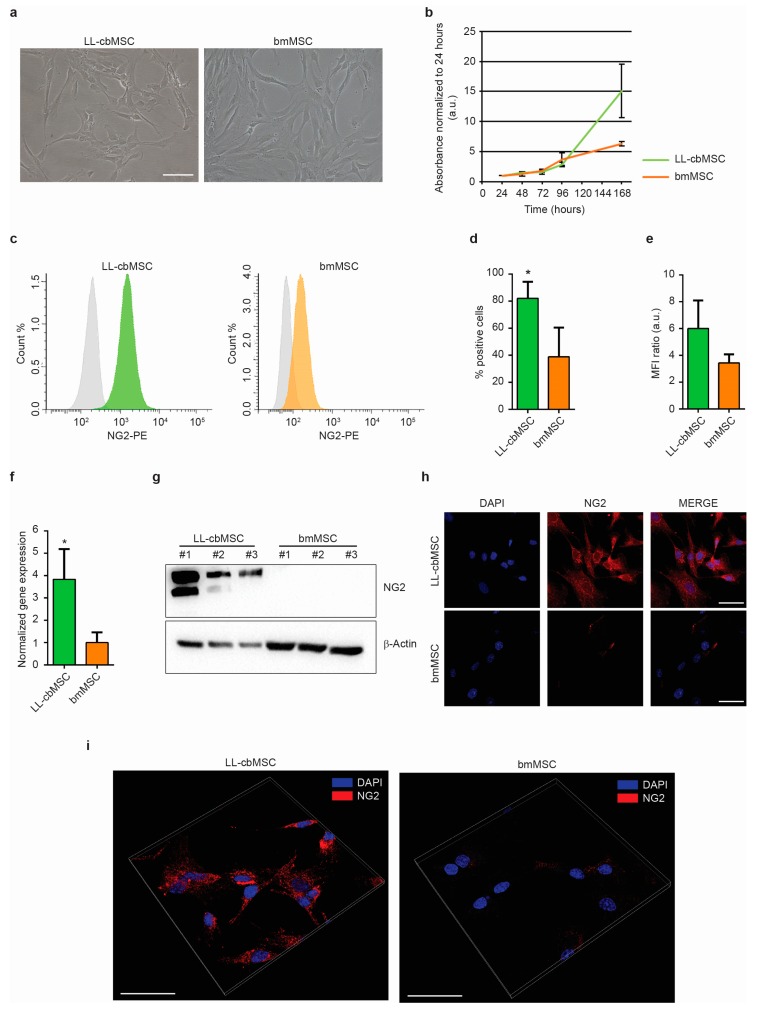
(**a**) Representative bright-field images of LL-cbMSC and bmMSC. Scale bar is 100 µm for both images. (**b**) Growth curve of LL-cbMSC and bmMSC. Mean and standard deviation are represented; *n* = 3 each experimental group; a.u., arbitrary units. (**c**) Representative flow cytometry histograms showing isotype (grey) and LL-cbMSC (green) or bmMSC (orange) NG2-PE fluorescent signal. (**d**) Histograms showing the percentage of NG2+ events measured by flow cytometry. Mean and standard deviation are represented; * *p* < 0.05; statistical analysis is two-tailed unpaired Student’s *t*-test, *n* = 3 each experimental group. (**e**) Histograms showing mean fluorescence intensity (MFI) ratios measured by flow cytometry. Mean and standard deviation are represented; *n* = 3 each experimental group. (**f**) Histograms showing NG2 gene expression measured by qPCR. Mean and standard deviation are represented; * *p* < 0.05; statistical analysis is two-tailed unpaired Student’s *t*-test, *n* = 3 each experimental group. (**g**) Upper panel: Western blot showing expression of the protein of interest. Lower panel: Western blot showing expression of a house-keeping protein. (**h**) Representative confocal microscopy images of NG2 expression compared to nuclei signal (DAPI). Scale bar is 50 µm for all images. (**i**) Representative confocal microscopy three-dimensional projections of NG2 protein localization compared to nuclei signal (DAPI). Scale bar is 50 µm for all images.

The expression of NG2 was addressed in long-living cord blood mesenchymal stem cells (LL-cbMSC) and bone marrow MSC (bmMSC) as representative of perinatal and adult MSC populations, respectively. Indeed, as shown in Figure 1a, LL-cbMSC recapitulated the perinatal MSC thin morphology (Figure 1a), and the high proliferation potential (Figure 1b) compared to bmMSC, as measured by an MTT assay. Furthermore, the population doubling time calculated during the exponential growth phase was 10.4 ± 1.8 h for LL-cbMSC and 17.6 ± 1.3 h for bmMSC (*p* = 0.0052; two-tailed unpaired Student’s *t*-test; *n* = 3 each experimental group).

The expression of NG2 at the extracellular leaflet of the plasma membrane was addressed by flow cytometry. Both LL-cbMSC and bmMSC stained positive compared to isotype controls, indicating a consistent presence of NG2 surface antigen (Figure 1c). The NG2 positivity was detected in almost all LL-cbMSC, compared to less than half bmMSC (Figure 1d). In addition, mean fluorescence intensity ratios indicated that NG2 relative abundance was higher in LL-cbMSC than bmMSC (Figure 1e). The significant difference in NG2 expression was confirmed by qPCR, which evidenced a 4-fold change in transcript level (Figure 1f). Relevant differences in NG2 expression were also determined by Western analysis, where bmMSC protein levels were undetectable (Figure 1g). Immunofluorescent images were also acquired, confirming stronger NG2 antigen expression in LL-cbMSC than bmMSC (Figure 1h). Cellular localization of NG2 protein was further investigated by three-dimensional projection. Fluorescent NG2 signal was observed at the cell membrane and cytoplasmic compartments (Figure 1i), consistent with the flow cytometry data.

### 3.2. Cord Blood and Bone Marrow Mesenchymal Stem Cells Produce Extracellular Vesicles Sharing Identity-Defining Features

**Figure 2:**
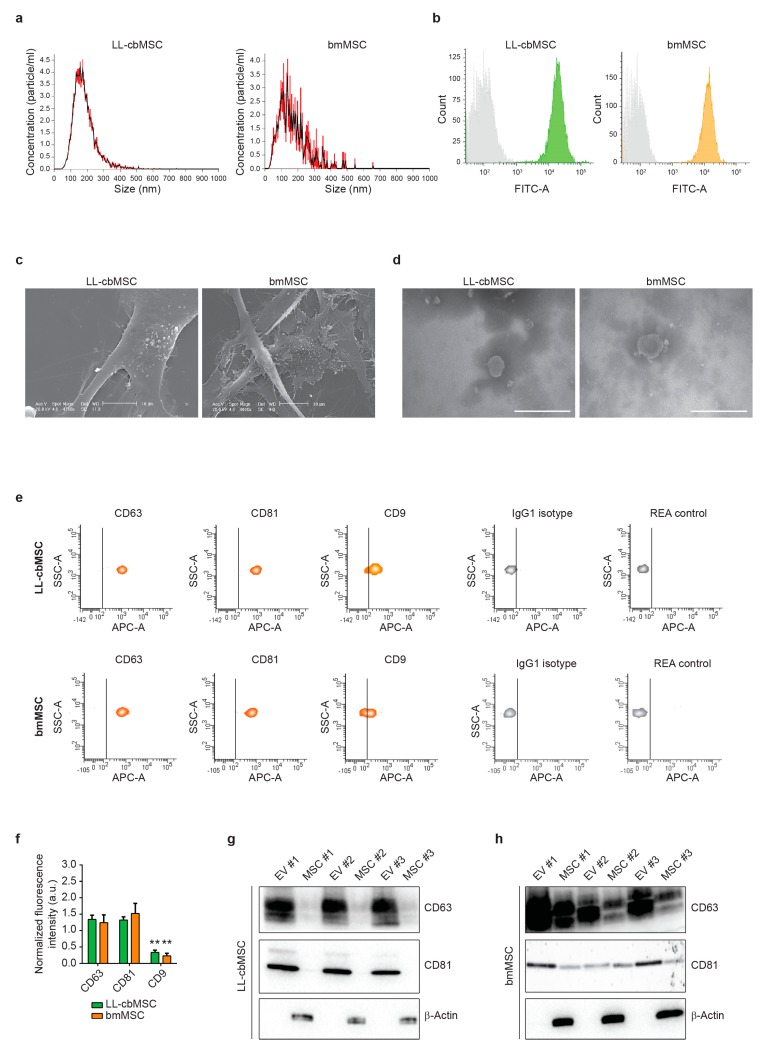
(**a**) Representative histograms of particle size distribution by nanoparticle tracking analysis. Mean and standard deviation of *n* = 5 repeated measures are represented. (**b**) Representative flow cytometry histograms showing isotype (grey) and LL-cbMSC (green) or bmMSC (orange) CFSE fluorescent signals. (**c**) Representative scanning electron microscopy images of extracellular vesicle (EV) budding by MSC. Scale bar is 10 µm for all images. (**d**) Representative transmission electron microscopy images of isolated EV. Scale bar is 500 µm for all images. (**e**) Representative flow cytometry density plots showing fluorescent signal as detected by APC-A channel for different antigens and controls; IgG1 isotype is CD63 and CD9 control; REA control is CD81 control. The vertical bar marks positivity for APC-A signal. (**f**) Histograms showing APC-A signal normalized to the average of mean fluorescence intensity of CD63, CD81, and CD9. Mean and standard deviation are represented, *n* = 3 for each experimental group; a.u., arbitrary units. (**g**,**h**) Western blots showing expression of the proteins of interest in EV and the respective parental cells for LL-cbMSC (**g**) and bmMSC (**h**).

To assess the generation of extracellular vesicles (EV) by LL-cbMSC and bmMSC, the respective cell culture supernatants were analyzed by nanoparticle tracking analysis (NTA). NTA showed similar EV size distribution profiles, mainly ranging from 50 to 300 nm (Figure 2a). Average mode particle size was 181 ± 59 nm for LL-cbMSC and 130 ± 40 nm for bmMSC (*p* = 0.285; two-tailed unpaired Student’s *t*-test; *n* = 3 each experimental group). Particle concentration was significantly higher (*p* = 0.028; two-tailed unpaired Student’s *t*-test) in LL-cbMSC (8.6 ± 4.1 × 10^9^ particle/mL) than bmMSC (0.6 ± 0.2 × 10^9^ particle/mL). 

To address whether the analyzed particles were membrane-enclosed cytoplasm-containing intact bodies, positivity for carboxyfluorescein succinimidyl ester (CFSE) staining was performed. Carboxyfluorescein diacetate succinimidyl ester (CFDA-SE) is the CFSE precursor able to diffuse through cell membranes thanks to its acetated groups. These are then removed by cytoplasmic esterares, which convert the molecule into an amine-reactive fluorescent ester that covalently binds mainly to lysine residues. MSC EV were analyzed by flow cytometry, and homogenous populations were selected (Supplementary Figure S1a) to detect CFSE signal (Figure 2b). Positive events were 91.0 ± 4.6% for LL-cbMSC and 93.5 ± 1.5% for bmMSC, showing no significant difference (*p* = 0.418; two-tailed unpaired Student’s *t*-test; *n* = 3 each experimental group). Electronic microscopy (EM) provided further confirmation of EV generation. Scanning EM showed budding of EV limited to small areas of the cell membrane (Figure 2c). Transmission EM confirmed similar MSC EV size and the presence of intact EV after isolation by ultracentrifugation (Figure 2d).

EV identity was then investigated, addressing the expression of typical exosomal tetraspanin markers. As shown in Figure 2e, bead-based flow cytometry allowed for the assessment of CD63, CD81, and CD9 positivity, compared to isotype controls. No significant differences (*p* > 0.05; two-tails unpaired Student’s *t*-test; *n* = 3 for each experimental group) were found between LL-cbMSC and bmMSC EV. Conversely, high statistical significance (*p* < 0.01; two-tails unpaired Student’s *t*-test; n = 3 for each experimental group) was found comparing CD63 and CD81 with CD9 for both LL-cbMSC and bmMSC EV (Figure 2f). The protein expression of tetraspanins was validated by Western analysis and also addressed in EV parental cells. Intriguingly, CD63 and CD81 abundance were higher in EV than the respective LL-cbMSC (Figure 2g) and bmMSC (Figure 2h) sources. Finally, protein expression of β-actin was addressed to assess EV preparation purity, since its presence is indicative of co-isolation of cell debris or apoptotic bodies containing cytoskeletal structures. The data showed detection of β-actin only in parental cells (Figure 2g,h), demonstrating the purity of released EV.

### 3.3. Parental Cell Differences in NG2 Abundance is Preserved in Respective Extracellular Vesicles

**Figure 3:**
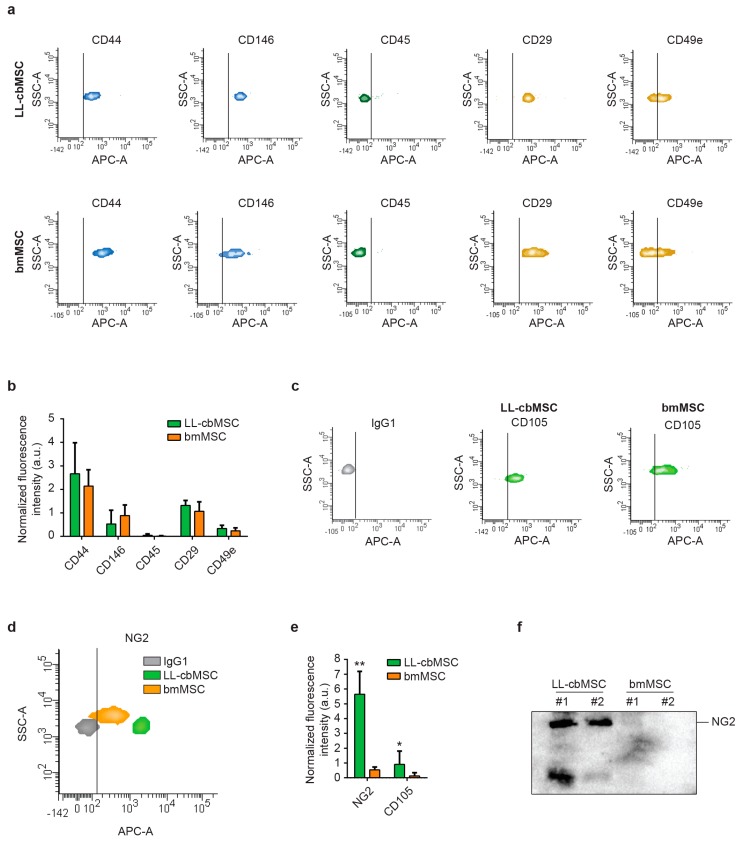
(**a**) Representative flow cytometry density plots showing fluorescent signal as detected by APC-A channel for different antigens and controls. The vertical bar marks positivity for APC-A signal. (**b**) Histograms showing APC-A signal normalized to the average of mean fluorescence intensity of CD63, CD81, and CD9. Mean and standard deviation are represented, *n* = 3 for each experimental group; a.u., arbitrary units. (**c**,**d**) Representative flow cytometry density plots showing fluorescent signal as detected by APC-A channel for CD105 (**c**), NG2 (**d**) compared to isotype IgG1 controls. The vertical bar marks positivity for APC-A signal. (**e**) Histograms showing APC-A signal normalized to the average of mean fluorescence intensity of CD63, CD81, and CD9. Mean and standard deviation are represented, *n* = 3 for each experimental group. (**f**) Western blot showing the expression of the protein of interest in extracellular vesicle samples.

Extended immunophenotyping was performed to investigate whether MSC EV preserved the surface antigen profile of parental cells. First, cell type-defining markers were considered (Figure 3a). Typical MSC markers (CD44, CD146) and integrins (CD29, CD49e) were found to be expressed at similar levels between LL-cbMSC and bmMSC EV (Figure 3b), whereas hematopoietic (CD45, CD14) and endothelial (CD31) markers were not expressed (Figure 3b and Supplementary Figure S1b). Strikingly, also both immune system-activating HLA-ABC and HLA-DRDPDQ were not detected (Supplementary Figure S1b). Furthermore, the CD105 MSC marker was found to be more expressed in LL-cbMSC than bmMSC EV, even though both signals were higher than the isotype control (Figure 3c). 

Second, we focused our attention on NG2 to address whether differential expression observed in parental LL-cbMSC and bmMSC was retained in released EV, thus investigating its possible use as MSC EV identity and quality marker. A remarkable difference was observed for NG2, for which an order of magnitude in fluorescence intensity distinguished isotype control from bmMSC EV, and bmMSC from LL-cbMSC EV (Figure 3d). These differences reached statistical significance after normalization on the mean of CD63, CD81, and CD9 mean fluorescence intensity (Figure 3e). To confirm that NG2 differences between LL-cbMSC and bmMSC are also preserved in the respective EV, Western analysis on isolated EV was performed. Consistent with flow cytometry results, NG2 protein expression was remarkably high in LL-cbMSC EV samples compared to undetectable signals from bmMSC EV samples (Figure 3f).

### 3.4. NG2-Rich Extracellular Vesicles Support the Growth of Human Endothelial Cells

The biological function of NG2 is still under investigation. Nevertheless, its role in the modulation of endothelial cell dynamics has already been defined [19,28]. In particular, extracellular or soluble NG2 was shown to either sequester endogenous angiogenesis inhibitors (i.e., angiostatin and endostatin) [29] or promote the action of endothelial growth factors (i.e., bFGF and PDGF-AA) [20,30]. To test EV-shuttled NG2 functionality, the proliferation of endothelial cells was measured after LL-cbMSC and bmMSC EV administration. Different experimental conditions were considered to address EV-shuttled NG2 action on bFGF and PDGF-AA pro-angiogenic effect and angiostatin inhibitory effect. In all experimental conditions, proliferation was estimated by automated real-time monitoring via the xCELLigence system. This system allowed for a fast and operator-independent determination of cell growth in live cell cultures without the use of dyes. Cell growth was estimated by increased cellular impedance measured by microelectrodes fused at the bottom of the culture surfaces.

**Figure 4:**
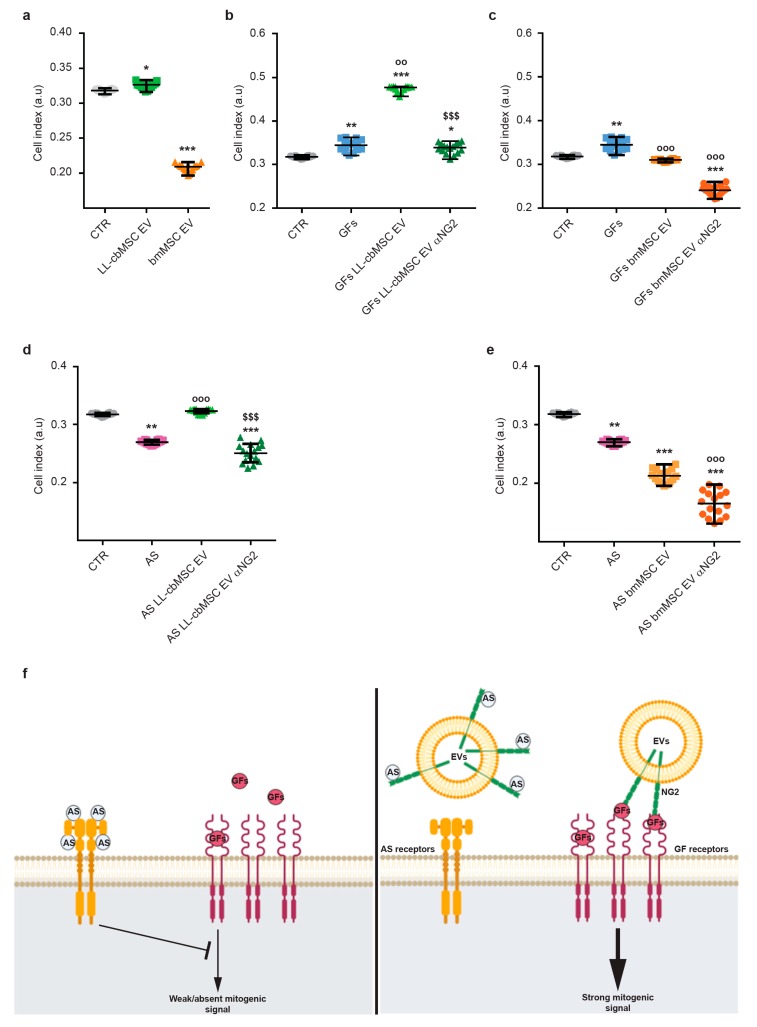
Scatter plots showing cell index values of endothelial cells measured by the xCELLigence system in the context of extracellular vesicles (EV) administration (**a**), LL-cbMSC (**b**) and bmMSC (**c**) EV effect on pro-angiogenic bFGF and PDGF-AA growth factors (GFs), LL-cbMSC (**d**) and bmMSC (**e**) EV effect on angiogenic inhibitor angiostatin (AS). (**f**) Schematic of EV-shuttled NG2 mechanisms of action on bFGF and PDGF-AA signaling and on angiostatin inhibitory effect to support endothelial cell growth. Statistical analysis was by Kruskal–Wallis non-parametric one-way ANOVA followed by Dunn’s multiple comparisons test; * *p* < 0.05, ** *p* < 0.01, *** *p* < 0.001, all vs. CTR condition; °° *p* < 0.05, °°° *p* < 0.05, all vs. GFs or AS conditions; ^§§§^ *p* < 0.05 vs. EV condition. a.u., arbitrary units; CTR, untreated endothelial cells; αNG2, NG2 antibody.

First, the effect of MSC EV administration on endothelial cells was investigated. LL-cbMSC EV elicited a significant stimulatory effect on cell growth compared to control endothelial cells, whereas bmMSC EV showed a dramatic inhibitory effect (Figure 4a). Second, the possible synergistic effect of concomitant administration of MSC EV and bFGF and PDGF-AA pro-angiogenic growth factors was addressed. An addition to culture media of bFGF and PDGF-AA induced a significant increase in the cell index (Figure 4b). Noteworthy, bFGF and PDGF-AA induction equaled the result observed in the previous experiment upon administration of LL-cbMSC EV alone (*p* = 0.1124; Mann–Whitney test). More importantly, the positive action of endothelial growth factors on cell growth was remarkably reinforced by LL-cbMSC EV administration and completely abrogated blocking NG2 antigen by the anti-NG2 antibody. Such effects were not observed upon bmMSC EV administration, where the growth factors-triggered increase was inhibited to control levels (Figure 4c). Moreover, the addition of the anti-NG2 antibody worsened this EV-driven profile. Third, the possible antagonistic action of MSC EV on angiostatin angiogenic inhibitor was studied. Strong inhibition of endothelial cell proliferation was elicited by angiostatin and nicely rescued by LL-cbMSC EV (Figure 4d). Again, this phenotype was utterly abolished only by LL-cbMSC EV administration. Importantly, the beneficial effect of LL-cbMSC EV was abrogated by the anti-NG2 antibody. Consistent with the previous results, bmMSC EV significantly amplified the inhibitory action of angiostatin, which was further exacerbated by anti-NG2 antibody administration.

## 4. Discussion

Clinical translation of innovative advanced therapy medicinal products (ATMP) entails a great effort dedicated to dealing with cellular derivatives as a proper pharmaceutical. Standardized and scalable processes are needed, along with appropriate and straightforward assays to define ATMP identity and quality. The exact classification of extracellular vesicles (EV) within or outside the ATMP framework is still under debate [14]. Nonetheless, it is highly reasonable that vesicular therapies will need similar identity and quality assays as those requested for cellular therapies [15]. With regard to terminology, other groups working in the clinical translation of vesicular therapies have proposed “small EV” as a more appropriate term to refer to EV released by MSC, by reason of their size profile compared to other classes of EV lacking the same therapeutic properties [31].

In this frame, we investigated the potential application of neural/glial antigen 2 (NG2) as a specific marker for long-living cord blood mesenchymal stem cells (LL-cbMSC) EV, which showed an average size compatible with the “small EV” classification introduced above. Interestingly, NG2 antigen abundance on EV membranes mirrored that of parental cell plasma membranes. In detail, LL-cbMSC and secreted EV showed much higher positivity for NG2 than bmMSC and bmMSC EV. NG2 was reported to modulate cell growth via intracellular pathways [32], in addition to more studied extracellular effects on angiogenesis, through the promotion of endothelial cell motility and proliferation [20,29,30,33]. Based on these properties and previous studies [34], we set up a simple and straightforward assay to address whether MSC EV-shuttled NG2 could elicit similar effects on endothelial cells in vitro. In correlation with the higher expression of NG2, LL-cbMSC EV, but not bmMSC EV, showed positive effects on endothelial cell growth. Possible mechanisms of action of soluble NG2 were proposed by others [29,30]. In detail, NG2 was shown to directly interact with kringle domains contained in plasminogen and its bioactive fragments plasmin and angiostatin, leading to sequestration of these proteins and modulation of their inhibitory effects on endothelial cells. Moreover, NG2 was also shown to harbor binding sites specific for bFGF and PDGF-AA growth factors, through which more efficient signaling by these mitogenic factors is achieved in target cells. Therefore, NG2 could be used as an indicator not only of LL-cbMSC EV identity but also of their quality, meant as the preservation of EV biological activity through all phases of isolation, production, and storage.

A similar approach, linking identity-defining EV antigen with quality of EV preparation, was adopted by Witwer et al. in the same context of MSC EV production [31]. In their work, they focused on CD73 surface antigen and its ecto-5-prime-nucleotidase enzymatic activity, which catalyzes the conversion of AMP to adenosine. This activity can be easily addressed by commercially available assays [35]. CD73 is also a key MSC surface marker, as stated by the International Society for Cellular Therapy [21,22]. These features allow the implementation of CD73 as sentinel to monitor protein denaturation and loss of enzyme activity during the preparation or storage of MSC EV for diverse clinical applications. 

Another marker found to be more expressed in LL-cbMSC than bmMSC was CD105 or endoglin, another type 1 glycoprotein shared between MSC and endothelial cells that forms part of the TGFβ receptor complex. The functional relevance of this surface protein on EV has been already studied by others in the context of metastasis, where CD105^+^ microvesicles generated by cancer cells triggered angiogenesis supporting the formation of a pre-metastatic niche [36]. Surprisingly, both class I and II HLA antigens were not expressed on MSC EV membranes. This result was unexpected because HLA-ABC is highly expressed in MSC [16], whereas HLA-DRDPDQ is only expressed by antigen-presenting cells. This feature of EV could be a remarkable advantage compared to parental MSC when considering the immune response potentially elicited by cellular therapies. Furthermore, this result suggests that there is an active mechanism sorting membrane proteins to MSC EV, or that HLA antigens are specifically excluded. Another relevant result concerned the expression of CD63, CD81, and CD9. These tetraspanins are the most well-known markers for EV, initially proposed to be specific for exosomes. Later studies showed a broader and more complex pattern of expression, extended to other classes of EV, including microvesicles [37,38]. MSC EV showed a robust and bright signal from CD63 and CD81, in contrast to a significantly dimmer CD9 signal. These data may indicate either a lower antigen density or a lower number of CD9^+^ MSC EV. Finally, CD63 and CD81 analyses clearly evidenced the enrichment of these antigens in EV compared to parental MSC. Thus, the use of CD63 and CD81 as broad identity markers for MSC EV could be evaluated.

In conclusion, we propose NG2 as surface antigen marker to specifically address LL-cbMSC EV identity and quality, thanks to its preferential expression in LL-cbMSC EV compared to bmMSC EV and to the retention of its functionality in shuttling EV. These features are required to set up consistent assays for pre-clinical and clinical studies, answering one of the urgent needs in the emergent field of MSC-based EV therapeutics.

## Supplementary Materials

The following are available online at https://www.mdpi.com/2073-4409/8/12/1524/s1, Figure S1: Flow cytometry analyses of MSC EV; Figure S2: Western blot analyses of MSC and MSC EV.
