# Supplementary material for: NG2 as an Identity and Quality Marker of Mesenchymal Stem Cell Extracellular Vesicles
Source: Cells. 2019 Nov 27;8(12):1524. doi: 10.3390/cells8121524 (PMC6953102; doi:10.3390/cells8121524)
Supplement: Supplementary file 1 [file cells-08-01524-s001.pdf]

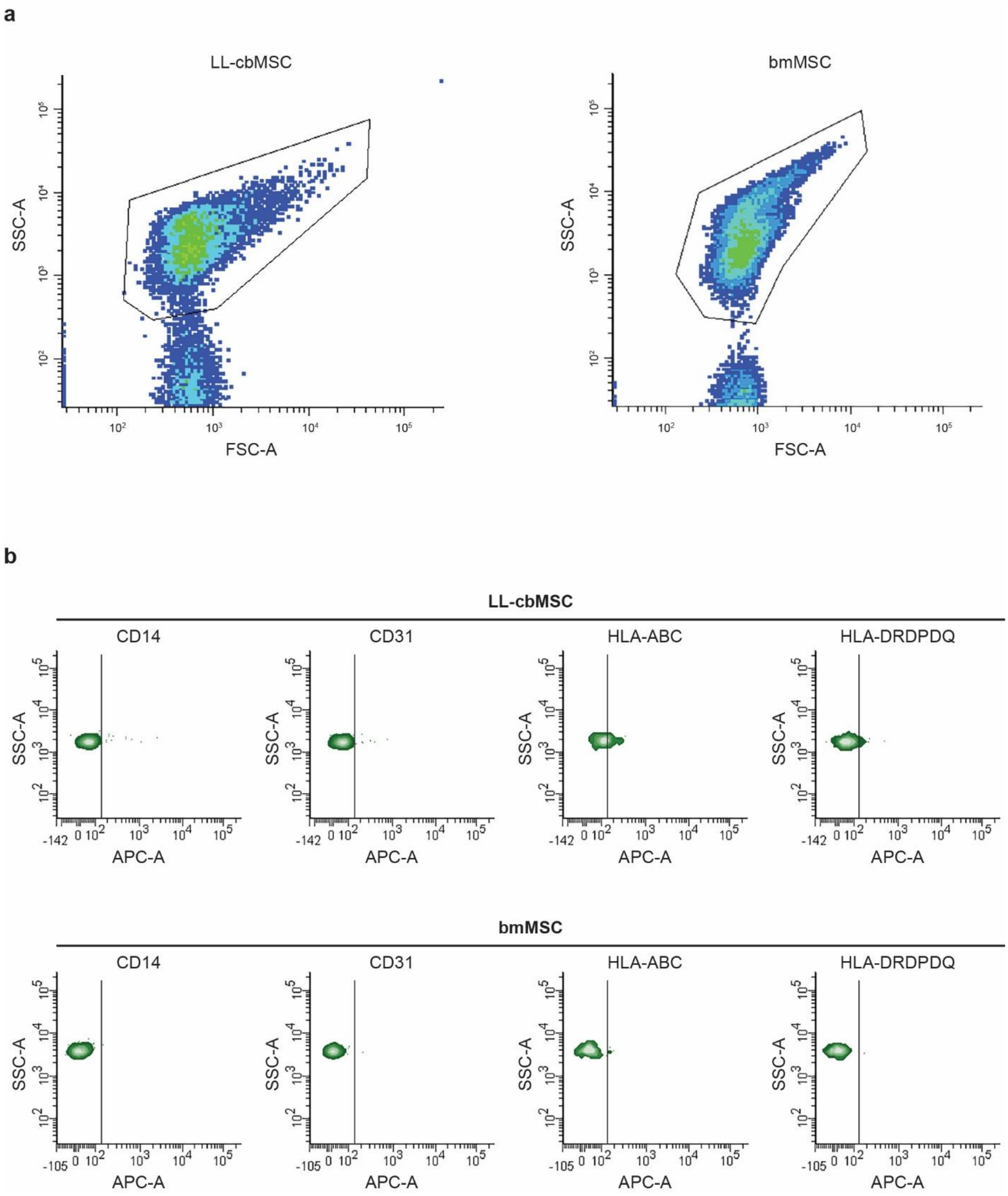

Supplementary Figure S1. Flow cytometry analyses of MSC EV. (**a**) Representative flow cytometry density plot showing morphological gate used to select extracellular vesicles for further analysis. (**b**) Representative flow cytometry density plots showing fluorescent signal as detected by APC-A channel for different antigens. The vertical bar marks positivity for APC-A signal.

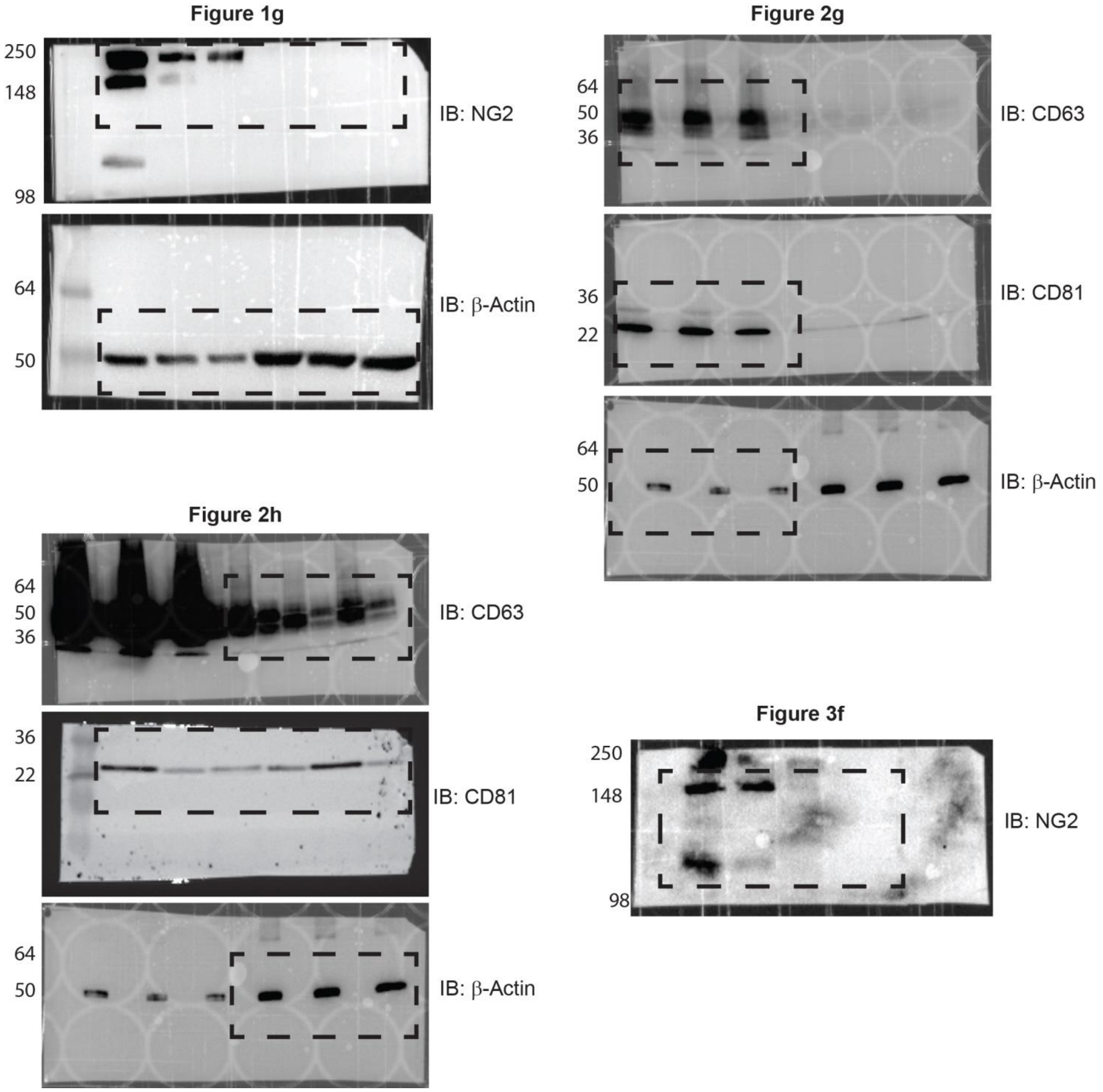

Supplementary Figure S2. Western blot analyses of MSC and MSC EV. Uncropped images of western analysis membranes showed in the specified main figures. Molecular weight marker bands are indicated. IB, immunoblot.
